# Pyoderma gangrenosum preceding the onset of extranodal natural killer/T-cell lymphoma: A case report

**DOI:** 10.1097/MD.0000000000004997

**Published:** 2016-10-07

**Authors:** Ting-Hua Yang, Chung-Hong Hu, Hsiou-Hsin Tsai

**Affiliations:** a Department of Dermatology, Taipei Medical University Hospital, Taipei, Taiwan; b Department of Dermatology, School of Medicine, College of Medicine, Taipei Medical University, Taipei, Taiwan.

**Keywords:** Epstein–Barr-encoding region, extranodal natural killer/T-cell lymphoma, pyoderma gangrenosum

## Abstract

**Introduction:**

Pyoderma gangrenosum (PG) is a neutrophilic dermatosis that may be associated with systemic diseases. The association of PG with lymphoid malignancies has rarely been reported. Extranodal natural killer/T-cell lymphoma (ENKTL) is a rare but aggressive entity with a poor prognosis. Here, we report the case of a patient who had idiopathic PG refractory to systemic steroids and subsequently developed ENKTL.

**Case report:**

A 70-year-old man presented with a 2-month history of intermittent fever and multifocal painful papules, plaques, and ulcerations on his extremities. The histological and culture results of the lesions were consistent with those of PG. A thorough work-up was performed and did not demonstrate any underlying systemic diseases including malignancy. The PG lesions were refractory to systemic steroid therapy. An enlarging nodule was observed over his right infraorbital area 4 months after the onset of the skin eruptions. The nodule was later biopsied and diagnosed as ENKTL by using histopathological and immunohistochemical studies. Fludeoxyglucose positron emission tomography/computed tomography revealed multiple intense fludeoxyglucose-avid masses in the bones and lungs, suggesting multiorgan metastases. The patient rejected chemotherapy and died 4 weeks after the diagnosis.

**Conclusion:**

The present case indicates that in any patient with idiopathic PG refractory to conventional therapy, the presence of any underlying disease or malignancy must be thoroughly evaluated. The present case serves as a reminder that when assessing patients with PG, clinicians should increase their awareness regarding the delayed association with malignancy, even in the absence of a concomitant systemic disease at presentation. Furthermore, the prompt evaluation of any suspicious lesions in the context of PG for the possibility of a malignant nature can improve the prognosis, particularly in cases of aggressive malignancy. Understanding the cutaneous spectrum of ENKTL is crucial because of its variable clinical appearance and aggressive nature. Our case demonstrates that PG can be a presenting sign of ENKTL.

## Abstract 2

Supplemental Digital Content is available in the text

## 1 Introduction

Pyoderma gangrenosum (PG) is a neutrophilic dermatosis that may be associated with systemic diseases such as arthritis, inflammatory bowel disease, hematologic dyscrasias, and malignancy. However, limited information is available regarding the association of PG with lymphoid malignancies. Here, we report the case of a patient who had idiopathic PG refractory to systemic steroid treatment and subsequently developed extranodal natural killer (NK)/T-cell lymphoma (ENKTL). The clinical spectrum of ENKTL and the pathogenesis underlying the association of PG with ENKTL are discussed.

## 2 Case report

**Figure 1:**
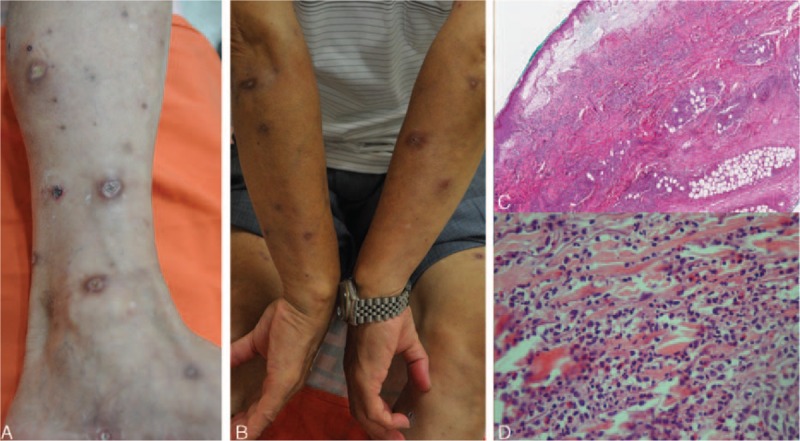
Pyoderma gangrenosum. A and B, Multiple painful and tender shallow ulcerations with an erythematous to violaceous border distributed on all extremities. C, Scanning view revealing prominent papillary edema and a dense inflammatory cell infiltrate involving the dermis and subcutaneous fat (hemotoxylin and eosin [H&E] 40×). D, Close-up view of inflammatory cells revealing numerous neutrophils and nuclear dust (H&E 400×). PG = pyoderma gangrenosum.

**Figure 2:**
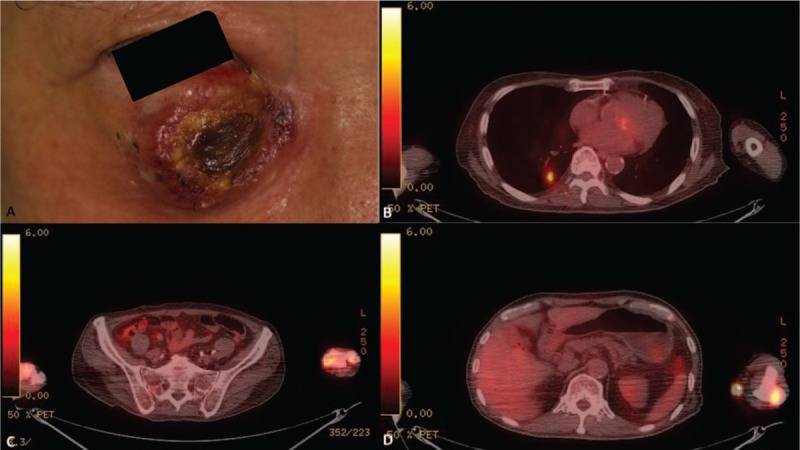
Extranodal natural killer/T-cell lymphoma. A, Enlarging nodule with central necrosis and a violaceous border on the patient's right infraorbital area. B, Selected axial PET/CT images exhibiting a nodular mass with intense FDG uptake in the right lower lung field. C, D, Selected axial PET/CT images showing extensive and marked FDG-avid sites in multiple sites of the bones and soft tissue of the left lower leg. Although the FDG uptake in soft tissue of the left lower leg may be consistent with the lesions of PG, other imaging findings were highly consistent with those of advanced ENKTL with widespread metastases in the body. ENKTL = extranodal natural killer/T-cell lymphoma, FDG = fludeoxyglucose, PET/CT = positron emission tomography/computed tomography, PG = pyoderma gangrenosum.

**Figure 3:**
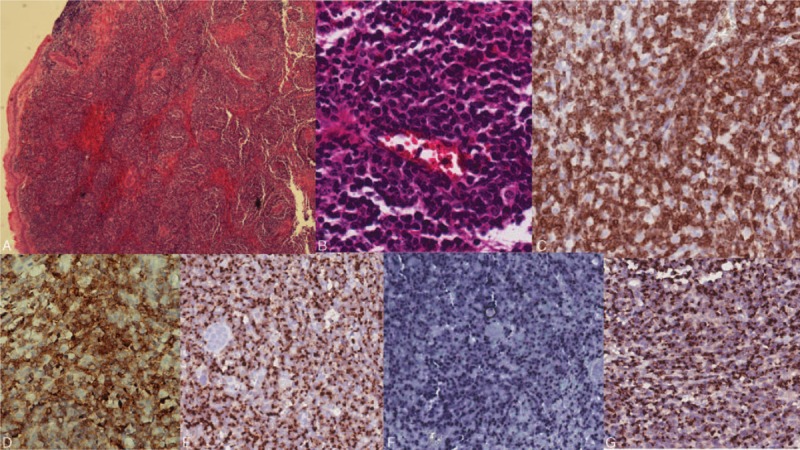
Extranodal natural killer/T-cell lymphoma—histopathology. A, Scanning view revealing a dense lymphoid infiltrate with a large area of geographic necrosis (hemotoxylin and eosin [H&E] 40×). B, High-power view showing prominent angioinvasion by tumor cells. The neoplastic cells are predominantly small to medium-sized, with irregular and elongate nuclear contours (H&E 400×). The neoplastic cells are diffusely positive for cytoplasmic CD3 (C), CD56 (D), granzyme B (E), and TIA-1 (F). G, The EBER ISH stain for EBV demonstrates diffuse positivity. EBER ISH = Epstein–Barr-encoding region in situ hybridization, EBV = Epstein–Barr virus, ENKTL = extranodal natural killer/T-cell lymphoma, TIA-1 = T-cell intracellular antigen-1.

A 70-year-old man presented to our clinic in August 2015 with a 2-month history of intermittent fever and multifocal painful erythematous and inflammatory papules and nodules, some of which had rapidly progressed into shallow ulcers surrounded by a zone of erythema. The eruptions were predominantly observed on his extremities (Fig. 1A and B). Although he had been treated with various systemic antibiotics in the past 2 months, the number of lesions still increased. On physical examination, no palpable lymphadenopathy, splenomegaly, or hepatomegaly was observed. Other symptoms and skin findings were unremarkable. He could not recall any drug use before the skin eruption or any exposure to halogens. Laboratory data revealed a low platelet count of 117 × 10^3^/μL (Supplementary Table 1). Other laboratory data including hemoglobin, C-reactive protein, cryoglobulin, creatinine, aminotransferase, C3, and C4 levels; white blood cell, neutrophil, and lymphocyte counts; antinuclear and antineutrophil cytoplasmic antibodies; and urine analysis parameters were normal. The bacterial cultures of ulcers were negative. We performed an incisional biopsy on one of the papules on his right dorsal hand; the patient reported that this papule had recently erupted, and the papule had a pseudovesicular and swollen appearance (Supplementary Fig. 1). The pathology revealed epidermal necrosis, prominent papillary edema, massive neutrophil infiltration in the entire dermis extending to the deep fat layer (Fig. 1C and D), and leukocytoclastic vasculitis involving the blood vessels of the subcutaneous fat (Supplementary Figs. 2–4). Neither granuloma formation nor any specific microorganism was observed under acid fast, Fite, Gram, and Periodic acid–Schiff stains. The clinicopathological appearance was compatible with that of PG, and immunohistochemistry (IHC) results were positive for CD3 and CD20, and negative for CD30 and CD56. These results were consistent with those of PG rather than cutaneous lymphoma. Therefore, a work-up for PG was conducted, including serum protein immunoelectrophoresis; serum IgA, IgG, and IgM level examination; chest x-ray; colonoscopy; whole-body computed tomography (CT); and positron emission tomography (PET). All these tests did not reveal any abnormalities or signs of occult malignancy. Hematological and chemistry panels were usually within normal limits during serial follow-ups, except for intermittent mild thrombocytopenia and an increased serum lactate dehydrogenase level of 322 IU/L. The patient received a diagnosis of idiopathic PG and was prescribed 30 mg of oral prednisolone every day along with topical steroids for the skin lesions during the subsequent 3 months. However, the skin lesions remained stationary, and an enlarging papule was observed over his right infraorbital area in October 2015, 4 months after the onset of the skin eruptions. Because the infraorbital lesion continued to enlarge into an erosive, oozing, and crusted nodule that was 3 cm in diameter (Fig. 2A), we performed a skin biopsy again on the lower periphery of the infraorbital lesion in December 2015. The histology revealed prominent geographic necrosis and dense pleomorphic tumor cells within the dermis with angioinvasion (Fig. 3A and B). IHC studies performed on the specimen revealed that neoplastic cells were positive for cytoplasmic CD3, granzyme B, T-cell intracellular antigen-1 (TIA-1), and CD56, but negative for CD4, CD5, CD8, CD20, CD21, PD1, cytokeratin, and CD34, indicating that the neoplastic cells belonged to NK/T-cell lineages. Epstein–Barr encoding region in situ hybridization was diffusely positive, and Ki-67 showed a positivity of 80% (Fig. 3C–G; Supplementary Figs. 5–9). Fiberscopy was performed to assess the disease status and revealed no gross tumor over the nasal cavity, larynx, and hypopharynx. Magnetic resonance imaging of the head and neck with and without contrast did not demonstrate the involvement of the nasal cavity. Fludeoxyglucose (FDG) PET/CT revealed multiple nodular lesions showing high FDG uptake in the right infraorbital area, limb bones, and both lungs (Fig. 2B–D). A bone marrow study was negative for lymphoma involvement. On the basis of the clinicopathological and immunophenotypic findings, the final diagnosis was nasal-type ENKTL, with multiorgan involvement including the skin, lungs, and bones. The patient rejected chemotherapy and received only radiotherapy to the cutaneous lesion on the right infraorbital area. He died 4 weeks after the initial diagnosis because of progressive pulmonary desaturation contributing to massive transfusion for hemodynamic instability complicated by pulmonary tumor progression.

Ethical approval was not required for this case report as it did not relate to the patient's privacy or treatment. Informed consent for the publication of this case report was obtained from the patient's wife because the patient had died.

## 3 Discussion

Extranodal natural killer/T-cell lymphoma is a rare type of cytotoxic lymphoma that is almost invariably associated with Epstein–Barr virus (EBV) and has a poor prognosis.^[1]^ It is subcategorized into “nasal” and “nasal-type” ENKTL on the basis of the primary sites of anatomical involvement. Nasal ENKTL primarily involves the nasal cavity and accounts for 60% to 90% of patients. Nasal-type ENKTL primarily involves extranasal sites and is highly aggressive, with a short survival and poor response to therapy.^[2–4]^

The composing malignant cells usually exhibit the NK-cell phenotype (IHC-positive for cytoplasmic CD3, CD56, and cytotoxic enzymes); however, a minority seems to be derived from cytotoxic T cells and has clonal T-cell receptor rearrangements with IHC positivity for surface CD3 and cytotoxic enzymes including perforin, granzyme B, and TIA-1.^[5]^ Because of the low incidence and nonspecific clinical appearance, ENKTL is very likely to be underdiagnosed or diagnosed late in the disease course. This may, in part, explain the low median survival rate after confirmation of the diagnosis reported in previous studies.^[6,7]^ The 2 major cutaneous manifestations of ENKTL are cellulitis and ulcers that are predominantly observed on the face and extremities, and they account for more than two-thirds of the clinical manifestations.^[2]^ Other clinical appearances that have been previously reported include plaques and nodules. ^[2,7]^ Understanding the cutaneous spectrum of ENKTL is crucial and may facilitate the early recognition of this extremely rare entity.

Pyoderma gangrenosum is a rare inflammatory disease of an unknown etiology characterized by sterile neutrophil infiltration of the skin. PG often occurs in patients who have other diseases such as arthritis, inflammatory bowel disease, hematologic dyscrasias, and malignancy.^[8]^ Although the association of PG with hematological diseases is well-documented, its association with lymphoid malignancy is extremely rare.^[9]^ Our patient received a clinical diagnosis of PG 4 months before the diagnosis of ENKTL, a finding that we believe was not previously reported.

The exact pathogenesis of PG and its relationship with hematological diseases remain to be elucidated. Some authors have suggested that increased levels of serum cytokines and chemoattractants have a role in the pathogenesis of PG in the setting of underlying hematological diseases.^[10,11]^ Masuda et al reported that increased production of interleukin (IL)-8 by the tumor cells of anaplastic large cell lymphoma may be a potent activator of neutrophils and may cause neutrophilic inflammation contributing to the pathogenesis of PG.^[11]^ Furthermore, the production of IL-8 and other chemoattractants by tumor cells in the serum of patients with PG was reported.^[12]^ IL-8 is a potent chemoattractant and neutrophil activator, and NK and T cells are 2 cellular sources of IL-8 production.^[8]^ In addition, Terunuma et al^[13]^ reported that the activation of NK cells and subsequent production of cytolytic effector cells affect neutrophils, enabling subsequent immune activation. Taken together, the aforementioned findings indicate that the altered clonality of the NK/T-cell lineage observed in our case may elicit overproduction of cytokines or chemoattranctants such as IL-8 and thus account for subsequent neutrophilic proliferation and aggregation.

The present case has some limitations. First, skin biopsy of a different PG skin lesion was not performed immediately after the lack of response to corticosteroids. In previous reports, cases have been initially misdiagnosed as PG, mostly because a biopsy was not performed or the work-up was incomplete.^[14]^ Although the clinical and pathological findings of the present case were compatible with those of PG and IHC studies did not provide evidence of lymphoma, a repeated biopsy should still ideally be performed for further confirmation or revision of the diagnosis. Second, the investigation of infraorbital ENKTL was delayed. Although the presentation of the lesion was apparently different from that of the previous lesions, a biopsy was not performed until 2 months later. The delay, mostly attributable to the reluctance of the patient, may have had a considerable effect on the outcomes for tumors with such an aggressive nature.

To the best of our knowledge, this is the first case report of an association of ENKTL with PG, possibly because of the rarity and the high prevalence of underdiagnosis of both entities. The present case indicates that in any patient with idiopathic PG refractory to conventional therapy, the presence of any underlying disease or malignancy must be thoroughly evaluated. In addition, a delay in the presentation of ENKTL 4 months after PG serves as a reminder that when assessing patients with PG, clinicians should increase their awareness regarding the delayed association with malignancy, even in the absence of a concomitant systemic disease at presentation. Furthermore, the prompt evaluation of any suspicious lesions in the context of PG for the possibility of a malignant nature can improve the prognosis, particularly in cases of aggressive malignancy.

## Supplementary Material

Supplemental Digital Content
