# Supplementary material for: Pyoderma gangrenosum preceding the onset of extranodal natural killer/T-cell lymphoma: A case report
Source: Medicine (Baltimore). 2016 Oct 7;95(40):e4997. doi: 10.1097/MD.0000000000004997 (PMC5059062; doi:10.1097/MD.0000000000004997)
Supplement: Supplemental Digital Content [file medi-95-e4997-s001.doc]

**Supplementary Figure 1.**


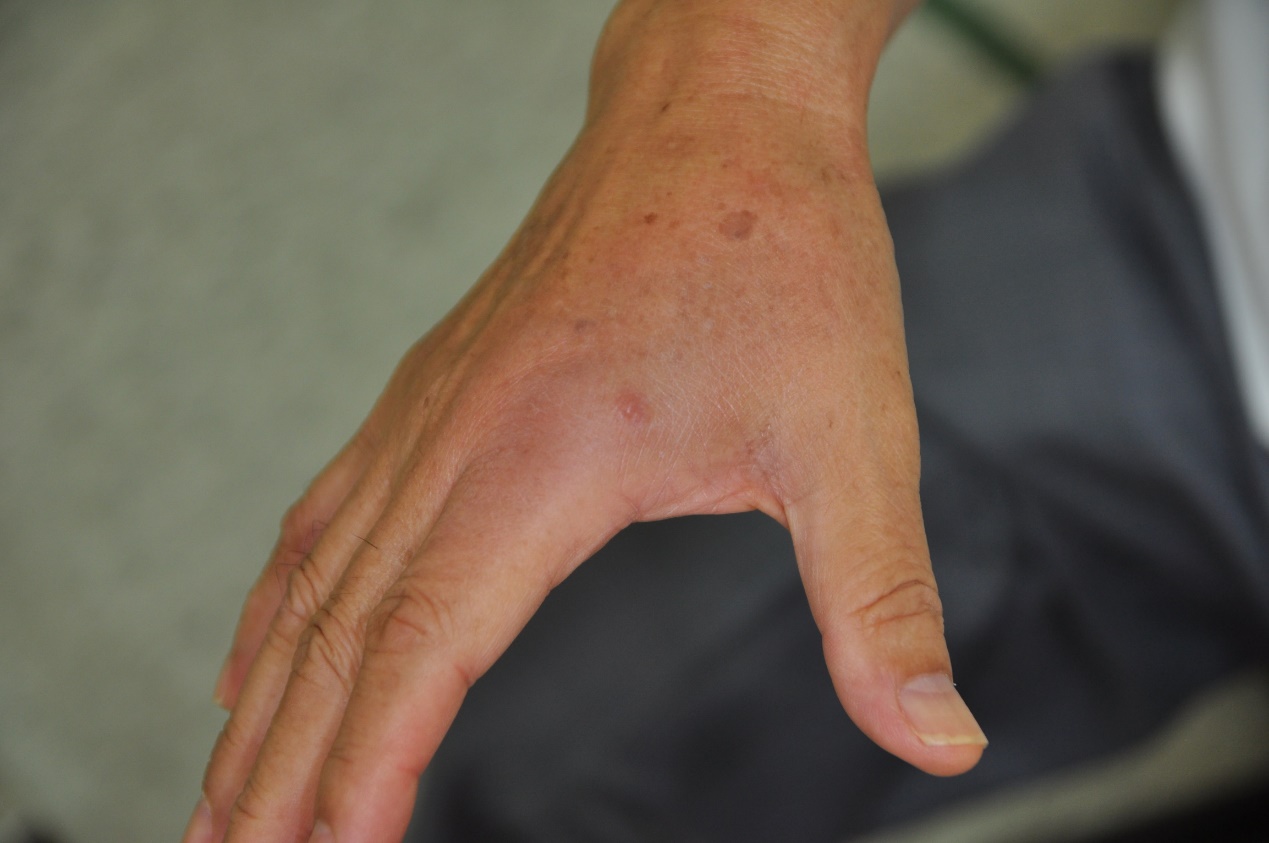


An incisional biopsy was performed on one of the papules on his right dorsal hand, which had recently erupted and showed a pseudovesicular appearance.

**Supplementary Figure 2.**


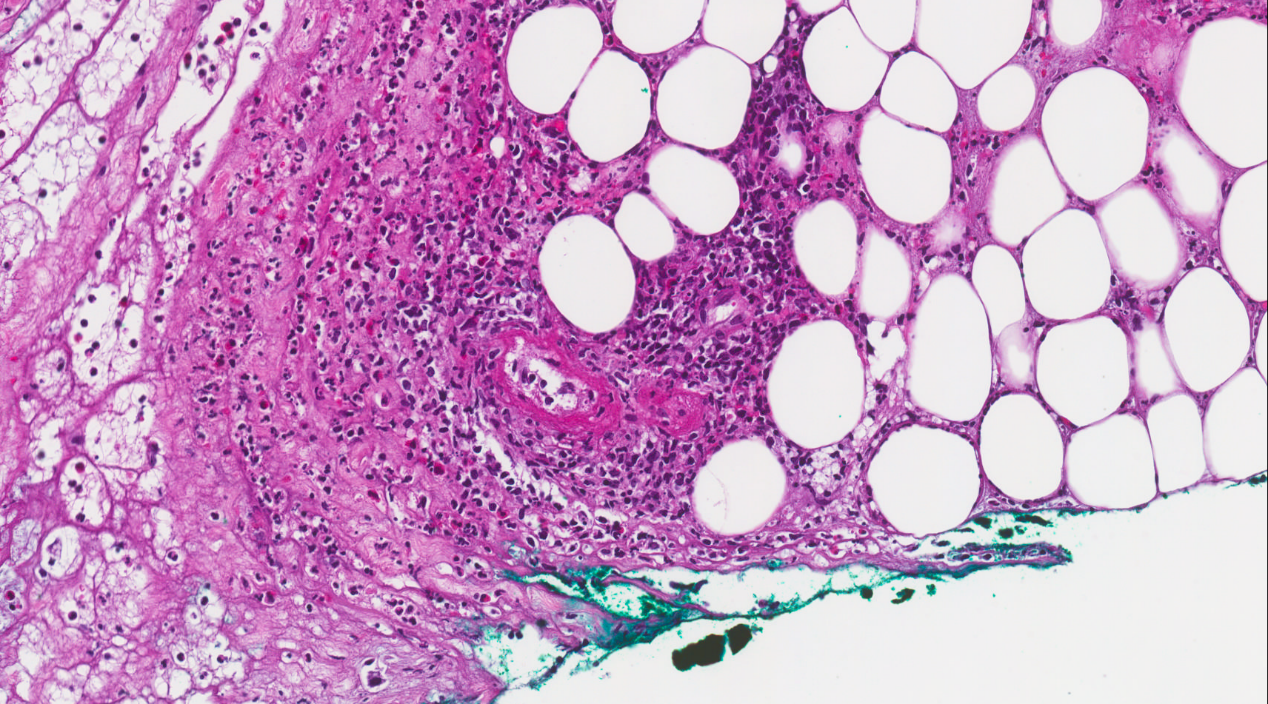


Pyoderma gangrenosum: The pathology revealed leukocytoclastic vasculitis involving the blood vessels of the subcutaneous fat.

**Supplementary Figure 3**


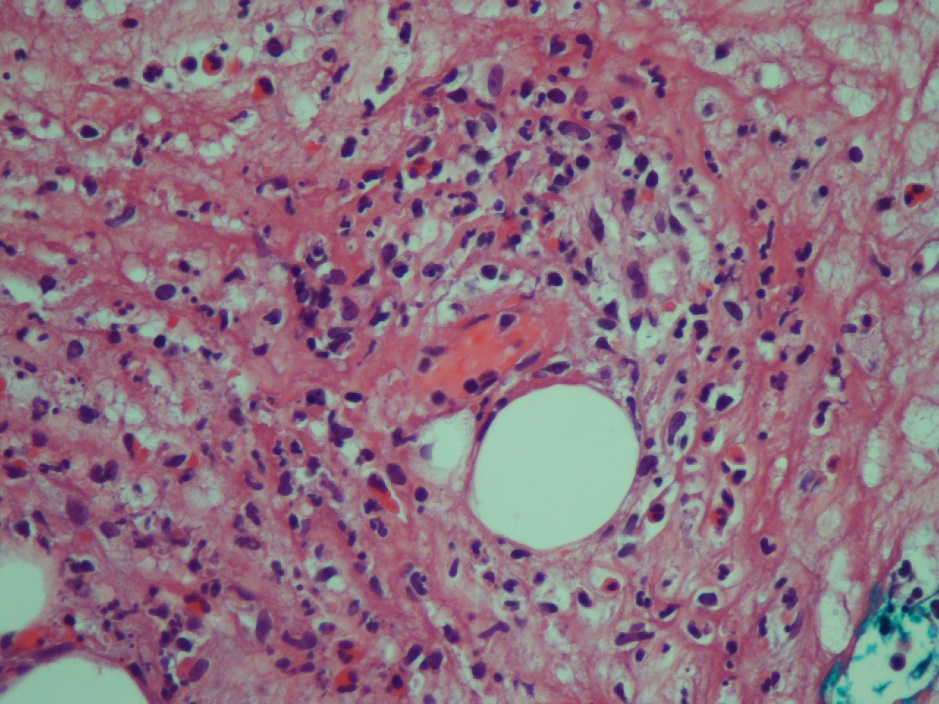


Pyoderma gangrenosum: The pathology revealed numerous neutrophils and nuclear dusts surrounding a vessel with prominent thrombus and fibrinoid degeneration of vessel wall.

**Supplementary Figure 4.**


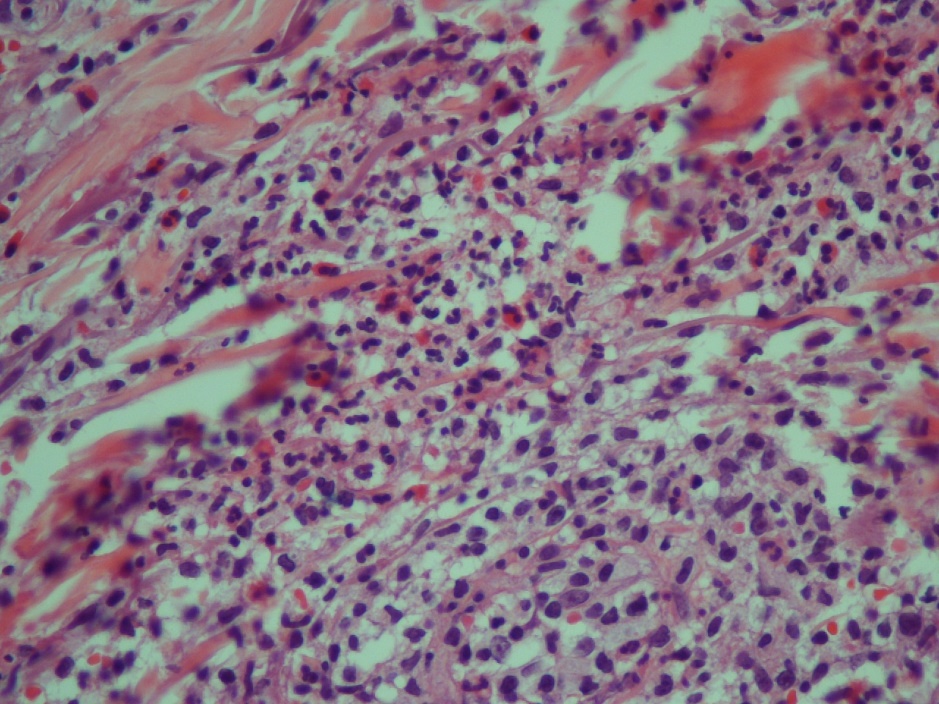


Pyoderma gangrenosum: This section showed dense neutrophil accumulation in the dermis.

**Supplementary Figure 5. Immunohistochemistry result for CD3**


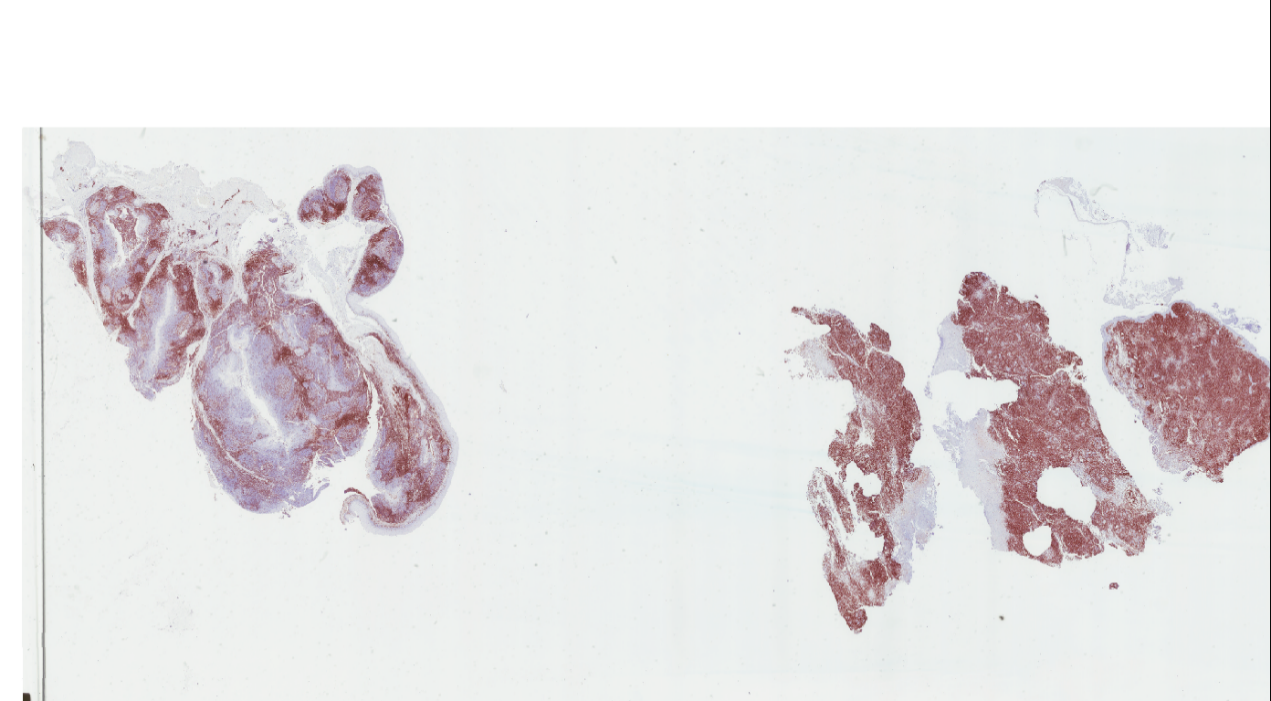


Extranodal NK/T cell lymphoma. The immunohistochemistry result for CD3 showed diffuse positivity (on the right side). The negative control is provided on the left side.

**Supplementary Figure 6. EBER ISH stain**

EBER


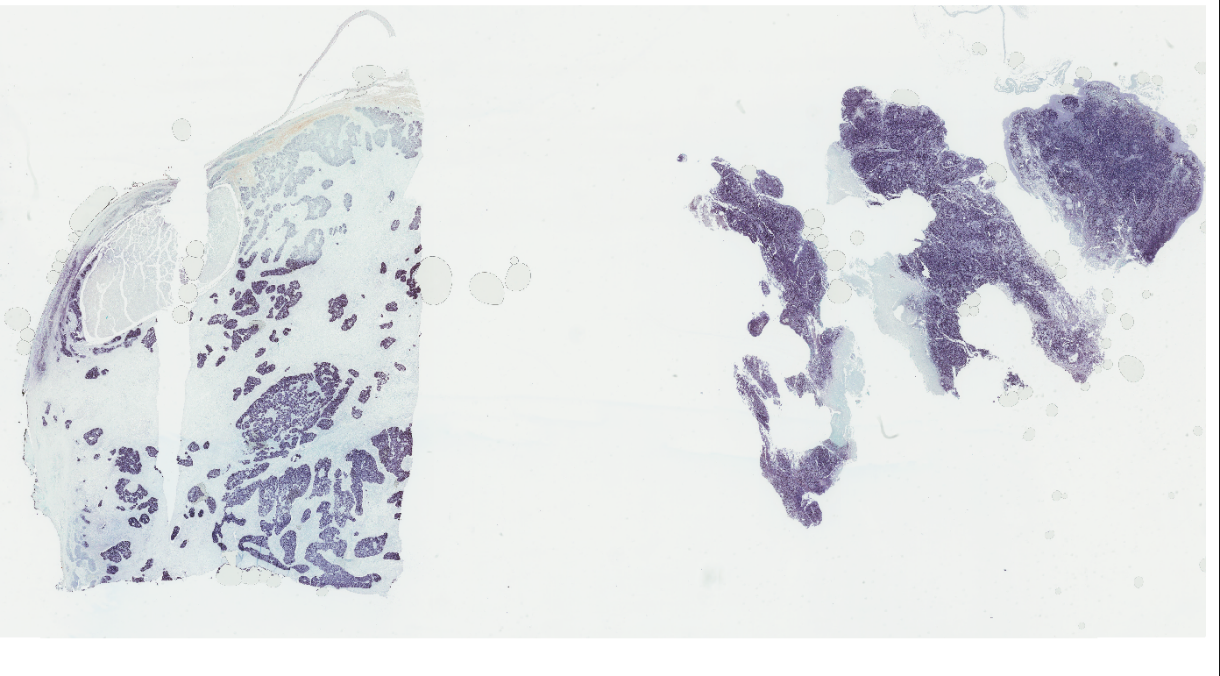


Extranodal NK/T cell lymphoma. The EBER ISH stain for EBV demonstrates diffuse positivity (on the right side). The negative control is provided on the left side.

EBER ISH = Epstein–Barr encoding region in situ hybridization; EBV= Epstein–Barr virus.

**Supplementary Figure 7.** **Immunohistochemistry result for TIA-1**


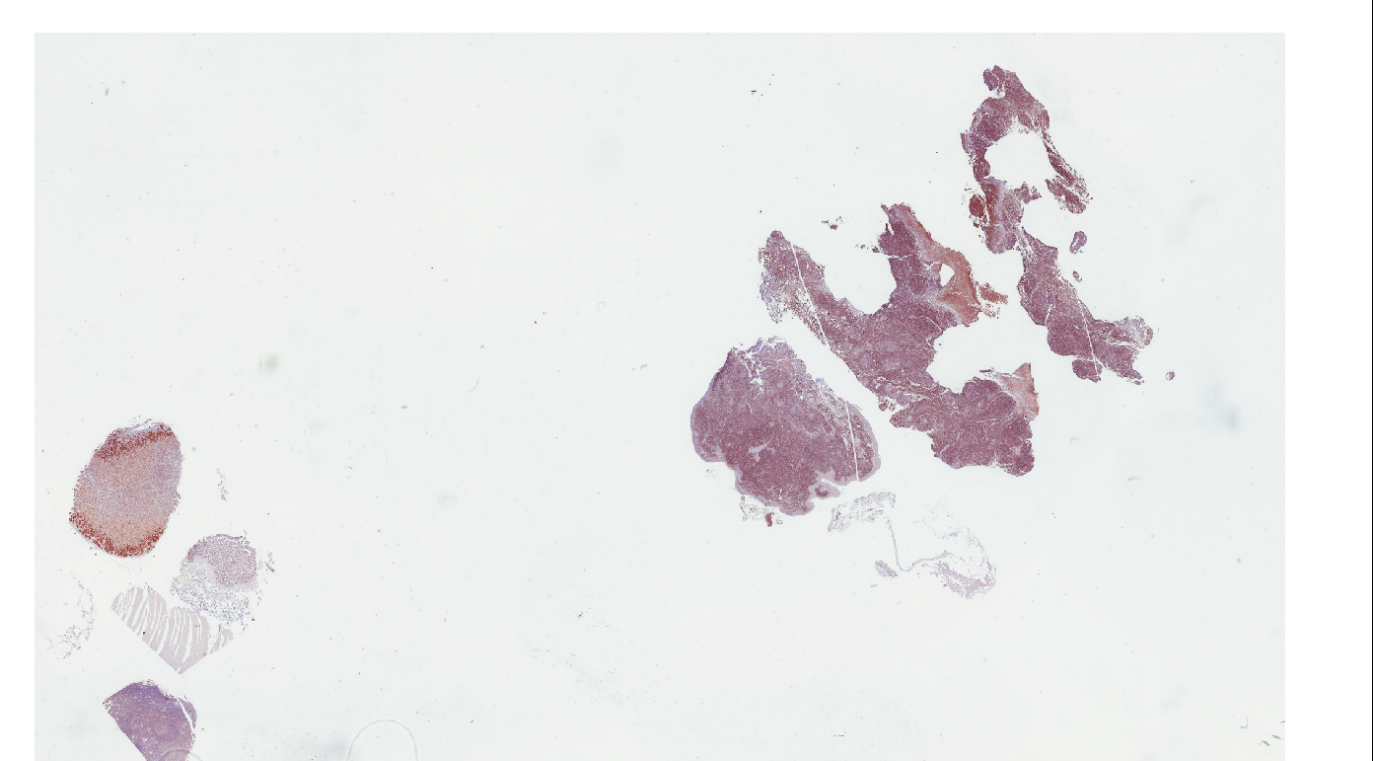


Extranodal NK/T cell lymphoma. The immunohistochemistry result for TIA-1 showed diffuse positivity (on the right side). The negative control is provided on the left side.

**Supplementary Figure 8**. Immunohistochemistry result for CD56

| 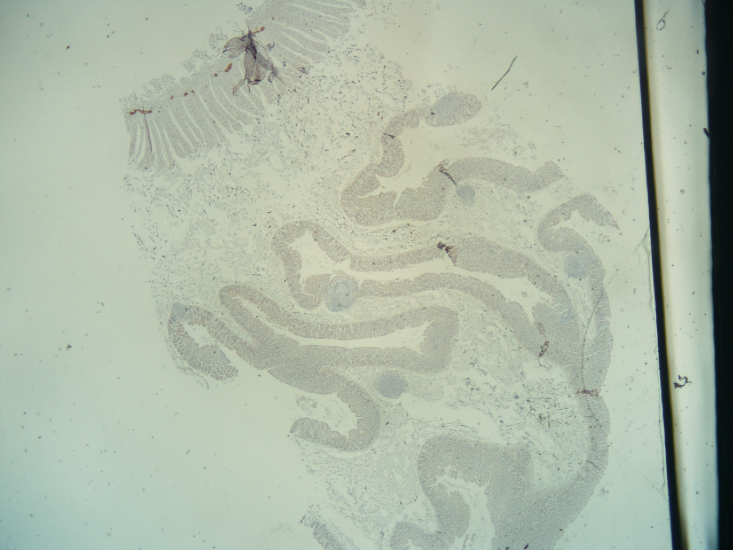 | 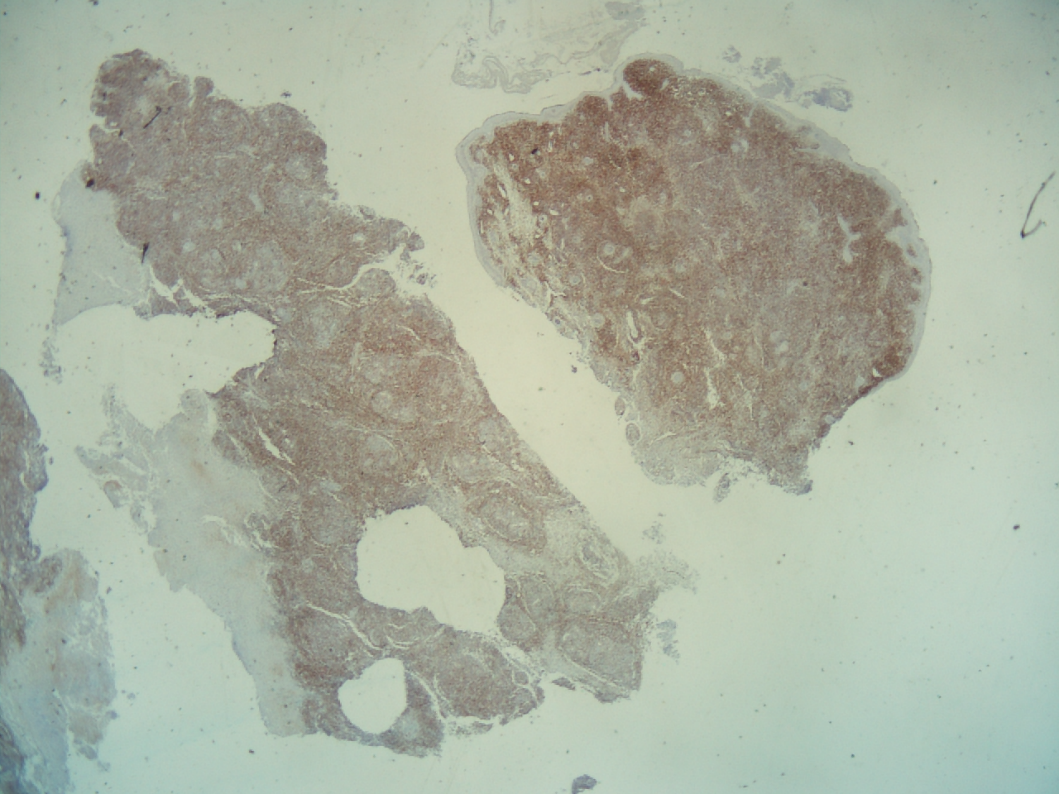 |
| --- | --- |

Extranodal NK/T cell lymphoma. The Immunohistochemistry result for CD56 showed diffuse positivity (on the right side). The negative control is provided on the left side.

**Supplementary Figure 9.** Immunohistochemistry result for Granzyme B


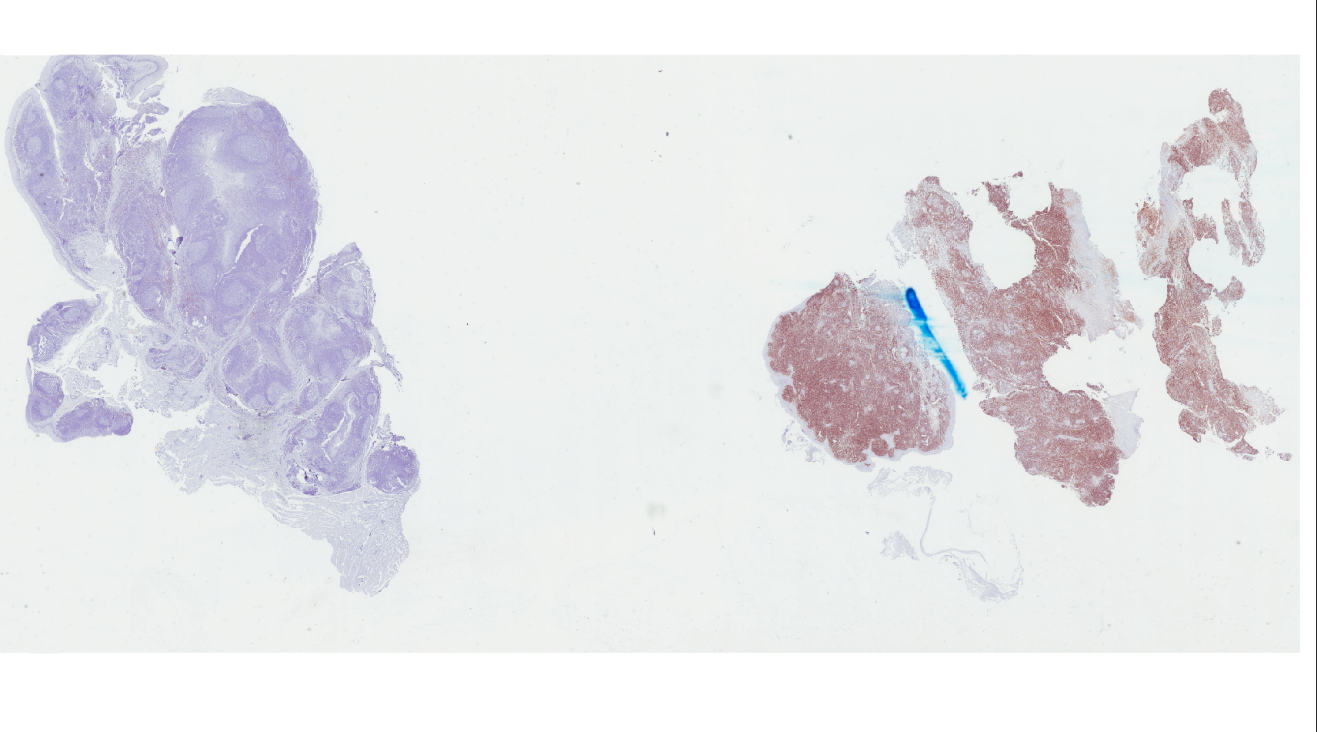


Extranodal NK/T cell lymphoma. The immunohistochemistry result for Granzyme B showed diffuse positivity (on the right side). The negative control is provided on the left side.

**Supplementary Table 1** Complete blood count when the patient was initially diagnosed as PG

| WBC | 8.97 10^3/uL [4.00-11.00] |
| --- | --- |
| RBC | 4.89 10^6/uL [4.20-6.10] |
| HGB | 15.4 g/dL [13.0-17.0] |
| HCT | 45.5 % [39.0-52.0] |
| MCV | 93.1 fL [80.0-99.0] |
| MCH | 31.4 pg [26.0-34.0] |
| MCHC | 33.8 g/dL [33.0-37.0] |
| RDW | 16.6 % [11.5-14.5] |
| PLT | 117 x10^3 /uL [130-400] |
| MPV | 6.69 fL [7.20-11.10] |
| RDW-SD | 53.8 fL |
| PDW | 16.7 fL |
| PCT | 0.08 % [0.16-0.35] |
| %NEUT | 58.9 % [40.0-74.0] |
| %LYM | 35.3 % [19.0-48.0] |
| %MONO | 5.6 % [2.0-12.0] |
| %EOS | 0.2 % [0.0-7.0] |
| %BASO | 0.0 % [0.0-2.0] |

**Supplementary Table 2** Laboratory data four months later when the patient was diagnosed with Extranodal NK/T cell lymphoma.

| WBC | 4.72 10^3/uL [4.00-11.00] |
| --- | --- |
| RBC | 3.37 10^6/uL [4.20-6.10] |
| HGB | 10.8 g/dL [13.0-17.0] |
| HCT | 31.1 % [39.0-52.0] |
| MCV | 92.2 fL [80.0-99.0] |
| MCH | 32.0 pg [26.0-34.0] |
| MCHC | 34.7 g/dL [33.0-37.0] |
| RDW | 18.4 % [11.5-14.5] |
| PLT | 96 x10^3 /uL [130-400] |
| MPV | 6.86 fL [7.20-11.10] |
| RDW-SD | 58.2 fL |
| PDW | 17.1 fL |
| PCT | 0.07 % [0.16-0.35] |
| %NEUT | 40.8 % [40.0-74.0] |
| %LYM | 48.4 % [19.0-48.0] |
| %MONO | 8.2 % [2.0-12.0] |
| %EOS | 1.3 % [0.0-7.0] |
| %BASO | 1.3 % [0.0-2.0] |
